# Hyperbaric Oxygen therapy for older adults with T2D and Mild Cognitive Impairment ‐ A baseline characteristics

**DOI:** 10.1002/alz.092679

**Published:** 2025-01-09

**Authors:** Ori Benari, Ramit Ravona‐Springer, Mary Sano, Barbara B. Bendlin, Abigail Livny, Ganit Almog, Derek Leroith, Michal S Beeri

**Affiliations:** ^1^ Tel Aviv University, Tel Aviv, HaMerkaz Israel; ^2^ The Joseph Sagol Neuroscience Center, Sheba Medical Center, Ramat Gan, Ha Merkaz Israel; ^3^ Tel Aviv University, Tel Aviv Israel; ^4^ Memory clinic, Sheba Medical Center, Tel Hashomer Israel; ^5^ The Joseph Sagol Neuroscience Center, Sheba Medical Center, Tel Hashomer Israel; ^6^ Icahn School of Medicine at Mount Sinai, New York, NY USA; ^7^ James J Peters VAMC, Bronx, NY USA; ^8^ Wisconsin Alzheimer’s Disease Research Center, University of Wisconsin‐Madison, School of Medicine and Public Health, Madison, WI USA; ^9^ Wisconsin Alzheimer’s Institute, University of Wisconsin School of Medicine and Public Health, Madison, WI USA; ^10^ Department of Diagnostic Imaging, Sheba Medical Center, Tel Hashomer Israel; ^11^ The Joseph Sagol Neuroscience Center, Sheba Medical Center, Ramat Gan Israel; ^12^ Herbert and Jackeline Krieger Klein Alzheimer’s Research Center, Rutgers Biomedical and Health Sciences, Newark, NJ USA

## Abstract

**Background:**

Hyperbaric oxygen therapy (HBOT) is a treatment in which oxygen‐enriched air (up to 100%) is administered to patients in a chamber at a pressure above one atmosphere absolute and is approved for the treatment of T2D ischemic wounds. Type 2 diabetes (T2D) is a risk factor for dementia. Ischemia due to vascular pathology is hypothesized to be an underlying mechanism for this association. Evidence small clinical trials suggests that HBOT improves hypoxic/ischemic brain injuries, consequently inducing brain angiogenesis, leading to cognitive improvement.

**Method:**

We have conducted a double blind, placebo controlled, clinical trial on brain and cognitive outcomes in elderly with T2D and mild cognitive impairment (MCI) to compare the effects of HBOT vs. sham. This analysis reports the baseline characteristics of those recruited.

**Result:**

A total of 155 participants, comprising 69% males (mean age 71.26) and 31% females (mean age 70.23), met the eligibility criteria to commence hyperbaric oxygen therapy (HBOT) with a Total Clinical Dementia Rating (CDR) of 0.5, Mini‐Mental State Examination (MMSE) score greater than 23, and no contraindications for HBOT. The average MMSE score was 28.5 for males and 28.4 for females. Beck’s Depression Inventory (BDI) revealed a mean score of 5.1 for males and 6.1 for females. Notably, 119 participants exhibited impairment in the CDR memory domain (CDR > 0) and other associated domains.

**Conclusion:**

Recent studies have indicated that hyperbaric oxygen therapy (HBOT) can stimulate neuroplasticity and enhance cognition in individuals recovering from strokes and traumatic brain injuries. However, the impact of HBOT on cognition in patients with Type 2 Diabetes (T2D) who are at a high risk of dementia remains to be established. The outcomes of this study, if successful, could furnish compelling evidence regarding the potential benefits of HBOT on brain function and cognition within this distinctive patient population.

